# Impact of Home-Based Antineoplastic Treatment Administration on Portuguese Patients: A Real-World Study of the ONConsigo Project

**DOI:** 10.7759/cureus.114901

**Published:** 2026-08-20

**Authors:** Francisca Abreu, Marta Silva, Lidia Toscano, Ana Conceição Cardoso, Manuela Morais, Ana Rita Fortunato, Cátia Barbosa, Alexandra Teixeira, Camila Coutinho, Ilda Faustino

**Affiliations:** 1 Department of Oncology, Unidade Local de Saude do Alto Ave, Guimarães, PRT; 2 School of Medicine, University of Minho, Braga, PRT; 3 Department of Pharmacy, Unidade Local de Saude do Alto Ave, Guimarães, PRT

**Keywords:** eortc qlq-c30, home-based oncology care, home-based systemic treatment, patient-centered care, portugal's healthcare, quality of life (qol)

## Abstract

Background: Cancer places a substantial burden on patients and healthcare systems, highlighting the need for patient-centered care models. Home-based oncology care has emerged as a strategy to improve quality of life (QoL). The ONConsigo project, implemented in Portugal in June 2023, delivers systemic cancer treatment at home.

Methods: This observational cohort study included patients enrolled in the ONConsigo program. QoL was assessed using the European Organisation for Research and Treatment of Cancer Quality of Life Questionnaire Core 30 (EORTC QLQ-C30) and the Personal Well-Being Index (PWI) at baseline and 2-6 months after treatment initiation. Paired comparisons were performed using the Wilcoxon signed-rank test, with p-values adjusted for multiple comparisons using the Benjamini-Hochberg false discovery rate (FDR) procedure. Statistical significance was set at p<0.05.

Results: Sixty-two patients were included (median age: 82 years; 51.6% male). Most of them had prostate (50%) or breast cancer (48.4%), and 61.3% were treated with palliative intent. Significant improvements were observed in treatment-related interference with family life, social life, and financial difficulties (all FDR-adjusted p=0.01). No significant changes were observed in the remaining questionnaire items after correction for multiple comparisons.

Conclusions: Home-based oncology care was associated with improvements in perceived global health status and in family, social, and financial dimensions of QoL, reducing treatment-related interference in daily life and decreasing financial burden. These findings support domiciliary antineoplastic therapy as a feasible patient-centered care model that warrants further evaluation in prospective controlled studies.

## Introduction

Cancer is a major global health concern, contributing significantly to morbidity, mortality, and healthcare costs. According to GLOBOCAN 2020 estimates, approximately 19.3 million new cancer cases and 10 million cancer-related deaths occurred worldwide in 2020 [1]. In Portugal, GLOBOCAN 2020 estimated 60,467 new cancer cases (incidence) and 30,168 cancer-related deaths in 2020, corresponding to an age-standardized incidence rate of 261.8 per 100,000 population [2].

Beyond direct treatment costs, cancer imposes substantial economic burden related to informal care provided by patients and caregivers. This increasing impact on individuals, families, and healthcare systems highlights the need for more effective, patient-centered care models [3-8].

The concept of "Oncology Hospital at Home" has emerged as an alternative care model, aiming to deliver hospital-level oncologic care in the patient's home. Initially developed in palliative settings, this model has expanded to include selected systemic therapies, promoting a patient-centered approach while maintaining safety and quality standards [4-7]. Several studies have also demonstrated the feasibility of delivering active cancer treatment in the home setting, with high levels of patient acceptance and satisfaction [9].

In Portugal, the ONConsigo project was implemented in June 2023 within the Oncology Department of Unidade Local de Saúde do Alto Ave as one of the first reported home-based oncology care initiatives in the Portuguese National Health Service. Evaluating its impact on patients' quality of life (QoL) is essential to determine its clinical value and support future implementation. Therefore, the aim of this study was to evaluate changes in QoL among patients enrolled in the ONConsigo home-based oncology program using the European Organisation for Research and Treatment of Cancer Quality of Life Questionnaire Core 30 (EORTC QLQ-C30) and the Personal Well-Being Index (PWI).

## Materials and methods

Study design and participants

This was an observational cohort study with both prospective and retrospective components, based on real-world data from the Medical Oncology Department of Hospital da Senhora da Oliveira, part of Unidade Local de Saúde do Alto Ave, in Guimarães, Portugal.As this was an exploratory real-world study, no formal a priori sample size calculation was performed. Instead, all consecutive eligible patients enrolled in the ONConsigo home-based oncology program during the study period were included, and the sample size was therefore determined by the number of eligible participants available. Eligible participants were required to be aged ≥18 years, reside within 30 km or 30 minutes of Hospital da Senhora da Oliveira (Guimarães, Vizela, or Fafe), have adequate home conditions and telephone access for communication with the care team, have a histologically confirmed malignancy, be clinically stable, have an Eastern Cooperative Oncology Group (ECOG) performance status of 0-2, and be receiving systemic therapy suitable for home administration that had previously been administered in hospital for at least two treatment cycles.

Patients receiving intravenous cytotoxic chemotherapy were excluded. Eligible treatments were required to have low risk of hypersensitivity reactions, no aerosolization risk, prolonged stability after preparation, and an infusion duration of no more than one hour. Therapies included subcutaneous, intramuscular, oral, and selected intravenous treatments with short infusion times.

Data collection and questionnaire administration were performed between October 2023 and August 2024.

Outcome measures

The primary outcome was QoL assessment using the EORTC QLQ-C30 (Version 3.0) and PWI [10,11]. Questionnaires were administered at baseline (prior to home-based treatment initiation) and at the first routine follow-up assessment performed between two and six months thereafter, reflecting the real-world clinical follow-up schedule of the program.

As questionnaire implementation began after project initiation, some baseline assessments were completed retrospectively based on patient recall. The EORTC QLQ-C30 evaluates functional domains, global health status, and symptom burden, whereas the PWI assesses subjective well-being across multiple life domains using a 0-10 scale.

For this exploratory analysis, EORTC QLQ-C30 responses were analyzed at the individual item level using raw item scores instead of the standard 0-100 transformed domain scores. However, the Social Functioning domain (items 26 and 27) was analyzed as a single multi-item scale score according to the official EORTC QLQ-C30 scoring manual.

Within-patient comparisons between baseline and follow-up assessments were performed to evaluate changes in QoL following inclusion in the program.

Statistical analysis

Statistical analyses were performed using IBM SPSS Statistics for Windows, Version 29.0 (IBM Corp., Armonk, New York, United States). Results are summarized as median (interquartile range (IQR)). As questionnaire responses were ordinal and paired (baseline and follow-up assessments for each patient), within-patient changes were analyzed using the Wilcoxon signed-rank test, applied separately to the EORTC QLQ-C30 and PWI items. No missing paired questionnaire data were observed. Therefore, all analyses were based on 62 paired observations. For each item, the standardized test statistic (Z), exact unadjusted two-tailed p-value, Benjamini-Hochberg-adjusted p-value, and effect size (r) are reported. Effect sizes were calculated as r=|Z|/√N using the effective N (number of discordant pairs), because the Wilcoxon signed-rank test excludes tied observations. For transparency, the number of tied pairs and the effective N are reported for each comparison. 

Effect sizes were interpreted as small (≥0.10), medium (≥0.30), or large (≥0.50). To account for multiple comparisons, exact unadjusted p-values were adjusted using the Benjamini-Hochberg false discovery rate (FDR) procedure, applied independently to the EORTC QLQ-C30 (30 items) and PWI (eight items). Statistical significance was defined as an adjusted p-value of <0.05.

As the interval between baseline and follow-up assessments varied across participants, its potential influence on the observed changes was explored using Spearman's rank correlation between the follow-up interval and the within-patient change in questionnaire scores.

Ethics

The study was approved by the Health Ethics Committee of Hospital da Senhora da Oliveira (approval number: 156/2023). Data confidentiality and anonymization were ensured in accordance with international ethical standards.

## Results

A total of 62 patients were included in the final analysis, all of whom completed both baseline and follow-up assessments. Data collection was conducted between October 2023 and August 2024. Among these, 40 patients (64.5%) completed both baseline and follow-up questionnaires retrospectively after home-based treatment initiation. Baseline and clinical characteristics are presented in Table 1.

**Table 1:** Baseline patient characteristics IQR: interquartile range; ECOG: Eastern Cooperative Oncology Group

Characteristic	Value
Age, median (IQR), years	82 (74-85)
Male sex, n (%)	32 (51.6)
Primary cancer location, n (%)	
Prostate	31 (50)
Breast	30 (48.4)
Urothelial	1 (1.6)
Treatment intent, n (%)	
Palliative	38 (61.3)
Curative	24 (38.7)
ECOG performance status, n (%)	
0	8 (12.9)
1	47 (75.8)
2	7 (11.3)

Treatment regimens included hormone therapy (aromatase inhibitors, selective estrogen receptor degraders (SERDs), androgen receptor inhibitors; 54.8%), luteinizing hormone-releasing hormone (LHRH) analogues/antagonists (53.2%), bone-targeted therapies (19.4%), monoclonal antibodies (14.5%), cyclin-dependent kinase (CDK) inhibitors (6.5%), oral chemotherapy (capecitabine and vinorelbine; 8.1%), and immunotherapy (3.2%), with some patients receiving multiple therapies concurrently.

Baseline and follow-up median scores, standardized test statistics (Z), effect sizes (r), and FDR-adjusted p-values for all questionnaire items are presented in Table 2 (EORTC QLQ-C30) and Table 3 (PWI).

**Table 2:** EORTC QLQ-C30 item scores at baseline and follow-up Data are presented as median (IQR). Q1-Q30 denote the individual EORTC QLQ-C30 items. Exact unadjusted and Benjamini-Hochberg-adjusted p-values are shown. P-values were calculated using the Wilcoxon signed-rank test for paired samples and adjusted for multiple comparisons using the Benjamini-Hochberg FDR procedure, applied within the EORTC QLQ-C30 scale. Z is the standardized test statistic; r is the effect size (r=|Z|/√N), calculated using the effective N (number of discordant pairs); and tied pairs and effective N are reported for each item. IQR: interquartile range; EORTC QLQ-C30: European Organisation for Research and Treatment of Cancer Quality of Life Questionnaire Core 30; FDR: false discovery rate

EORTC QLQ-C30 item	Before home care median (IQR)	After home care median (IQR)	Negative ranks	Positive ranks	Ties	Valid n	Unadjusted p-value	FDR-adjusted p-value	Z statistic	Effect size (r; n =62)	Effect size (r, valid n)	Effect size magnitude
Strenuous activities (Q1)	4 (2)	4 (1)	7	10	45	17	0.194	0.448	-1.299	0.165	0.315	Medium
Long walk (Q2)	4 (2)	4 (2)	5	7	50	12	0.497	0.733	-0.679	0.086	0.196	Small
Short walk (Q3)	2 (2)	2 (2)	11	6	45	17	0.310	0.499	-1.015	0.129	0.246	Small
Need to stay in bed and chair (Q4)	1 (1)	1 (1)	7	5	50	12	0.261	0.473	-1.125	0.143	0.325	Medium
Need help with self-care (Q5)	1 (2)	1 (2)	3	9	50	12	0.128	0.343	-1.524	0.194	0.44	Medium
Limitations in work or daily activities (Q6)	3 (2)	3 (2)	7	11	44	18	0.556	0.733	-0.589	0.075	0.139	Small
Limitations in hobbies or leisure activities (Q7)	3 (2)	3 (2)	7	9	46	16	0.895	0.927	-0.132	0.017	0.033	Small
Shortness of breath (Q8)	1 (1)	1 (1)	5	14	43	19	0.022	0.180	-2.284	0.29	0.524	Large
Pain (Q9)	2 (2)	2 (3)	11	15	36	26	0.937	0.937	-0.079	0.01	0.015	Small
Need to rest (Q10)	3 (2)	3 (2)	9	7	46	16	0.294	0.499	-1.049	0.133	0.262	Small
Sleep difficulties (Q11)	2 (2)	2 (2)	7	6	49	13	0.827	0.913	-0.218	0.028	0.06	Small
Weakness (Q12)	2 (3)	2 (2)	11	12	39	23	0.850	0.913	-0.189	0.024	0.039	Small
Lack of appetite (Q13)	1 (1)	1 (0)	8	1	53	9	0.031	0.180	-2.161	0.274	0.72	Large
Nausea (Q14)	1 (0)	1 (0)	10	4	48	14	0.229	0.473	-1.204	0.153	0.322	Medium
Vomiting (Q15)	1 (0)	1 (0)	4	3	55	7	0.705	0.842	-0.378	0.048	0.143	Small
Constipation (Q16)	1 (0)	1 (0)	9	4	49	13	0.083	0.301	-1.734	0.22	0.481	Medium
Diarrhea (Q17)	1 (0)	1 (0)	8	2	52	10	0.130	0.343	-1.515	0.192	0.479	Medium
Tiredness (Q18)	2 (3)	2 (3)	10	10	42	20	0.726	0.842	-0.351	0.045	0.078	Small
Pain interference with daily activities (Q19)	2 (3)	2 (2)	13	7	42	20	0.054	0.261	-1.928	0.245	0.431	Medium
Difficulty concentrating (Q20)	1 (1)	1 (0)	8	1	53	9	0.075	0.301	-1.78	0.226	0.593	Large
Feeling tense (Q21)	1.5 (2)	1 (2)	3	6	53	9	0.250	0.473	-1.15	0.146	0.383	Medium
Feeling worried (Q22)	2 (2)	2 (2)	9	6	47	15	0.201	0.448	-1.279	0.162	0.33	Medium
Feeling irritable (Q23)	1 (1)	1 (1)	6	9	47	15	0.582	0.734	-0.551	0.07	0.142	Small
Feeling depressed (Q24)	2 (1)	2 (2)	10	13	39	23	0.551	0.733	-0.597	0.076	0.124	Small
Difficulty remembering things (Q25)	1 (1)	1 (2)	5	11	46	16	0.101	0.325	-1.641	0.208	0.41	Medium
Social functioning scale (Q26 and Q27)	5 (4)	2 (0)	40	1	21	41	5.385×10^-^⁸	7.808×10^-^⁷	-5.438	0.691	0.849	Large
Financial difficulties (Q28)	2 (2)	1 (0)	38	0	24	38	4.494×10^-^⁸	7.808×10^-^⁷	-5.47	0.695	0.887	Large
Overall quality of life (Q29)	4 (2)	4 (1)	8	18	36	26	0.027	0.180	-2.212	0.281	0.434	Medium
Overall health (Q30)	4 (2)	4 (1)	13	10	39	23	0.523	0.733	-0.639	0.081	0.133	Small

**Table 3:** PWI item scores at baseline and follow-up Data are presented as median (IQR). PWI1.1-PWI1.7 and PWI2 denote the individual items of the PWI. Exact unadjusted and Benjamini-Hochberg-adjusted p-values are shown. P-values were calculated using the Wilcoxon signed-rank test for paired samples and adjusted for multiple comparisons using the Benjamini-Hochberg FDR procedure, applied within the PWI. Z is the standardized test statistic; r is the effect size (r=|Z|/√N), calculated using the effective N (number of discordant pairs); and tied pairs and effective N are reported for each item. IQR: interquartile range; PWI: Personal Well-Being Index; FDR: false discovery rate

PWI item	Before home care median (IQR)	After home care median (IQR)	Negative ranks	Positive ranks	Ties	Valid n	Unadjusted p-value	FDR-adjusted p-value	Z statistic	Effect size (r; n=62)	Effect size (r, valid n)	Effect size magnitude
Standard of living (PWI1.1)	5.5 (3)	6 (3)	9	12	41	21	0.350	0.700	-0.934	0.119	0.204	Small
Health (PWI1.2)	6 (4)	7 (4)	4	10	48	14	0.031	0.124	-2.156	0.274	0.576	Large
Achieving in life (PWI1.3)	5 (2)	5 (2)	8	17	37	25	0.021	0.124	-2.307	0.293	0.461	Medium
Personal relationships (PWI1.4)	7.5 (5)	8 (5)	8	11	43	19	0.273	0.700	-1.096	0.139	0.251	Small
Personal safety (PWI1.5)	10 (2)	10 (1)	5	8	49	13	0.569	0.759	-0.569	0.072	0.158	Small
Community connectedness (PWI1.6)	8 (5)	8 (5)	11	7	44	18	0.930	0.930	-0.087	0.011	0.021	Small
Future security (PWI1.7)	10 (2)	10 (2)	7	7	48	14	0.680	0.777	-0.412	0.052	0.11	Small
Life as a whole (PWI2)	5 (2)	5 (2)	7	9	46	16	0.546	0.759	-0.603	0.077	0.151	Small

Six EORTC QLQ-C30 items reached nominal statistical significance before multiple-testing correction: shortness of breath (Q8), lack of appetite (Q13), family life interference (Q26), social life interference (Q27), financial difficulties (Q28), and overall quality of life (Q29) (all unadjusted p<0.05). After Benjamini-Hochberg adjustment, only family life interference (Q26), social life interference (Q27), and financial difficulties (Q28) remained statistically significant (all adjusted p=0.01). In addition, the pre-specified Social Functioning scale (items 26 and 27 combined) improved significantly from baseline to follow-up (scale score, Z=-5.44; exact p=5.4×10^-^⁸; adjusted p=7.8×10⁻⁷; 41 discordant pairs, r=0.85). Financial difficulties also improved (Z=-5.47; exact p=4.5×10⁻⁸; adjusted p=7.8×10⁻⁷; 38 discordant pairs, r=0.89). These findings were accompanied by reductions in median scores from baseline to follow-up (family life interference: 3 to 1; social life interference: 3 to 1; financial difficulties: 2 to 1). Shortness of breath (Q8), lack of appetite (Q13), and overall quality of life (Q29) were no longer statistically significant after adjustment (adjusted p=0.155) and demonstrated small effect sizes (r≤0.29). No significant changes were observed in the remaining EORTC QLQ-C30 items.

Two PWI items, satisfaction with health (PWI1.2) and overall life satisfaction (PWI2), reached nominal statistical significance before correction (unadjusted p=0.031 and p=0.021, respectively). Neither remained statistically significant after Benjamini-Hochberg adjustment (both adjusted p=0.124), and all PWI items demonstrated small effect sizes (r≤0.29). No PWI item showed a statistically significant change after adjustment.

The interval between assessments was not significantly correlated with the magnitude of change in any of the EORTC QLQ-C30 items that remained statistically significant after adjustment (Spearman's ρ=-0.03 and p=0.829 for family life interference (Q26); ρ=0.06 and p=0.689 for social life interference (Q27); ρ=-0.22 and p=0.117 for financial difficulties (Q28)), suggesting that variability in the follow-up interval did not materially influence the observed results.

## Discussion

The definition and assessment of QoL remain inherently challenging due to its subjective, individual, and multifactorial nature. Patients' perceptions are influenced by multiple variables, including underlying disease, treatment-related factors, and comorbidities, particularly in elderly populations [6,7].

Consistent with findings reported by Bordonaro et al. [9], which demonstrated improvements in several functional and symptom scales of the EORTC QLQ-C30 following home care implementation, our study identified statistically significant improvements in interference with family life, interference with social life, and financial difficulties. These findings suggest that domiciliary oncological care may positively influence selected aspects of QoL, particularly those related to patients' social functioning and socioeconomic burden.

The observed improvements in social functioning may be explained by the reduced disruption associated with avoiding repeated hospital visits, allowing patients to maintain greater participation in family and social activities. However, given the observational design, these explanations remain speculative. Although symptom-related items did not improve significantly after correction for multiple comparisons, home-based care may still reduce the practical burden of treatment by minimizing travel and hospital attendance [6].

From an economic perspective, the benefits of home-based care are particularly relevant for both patients and caregivers. Transportation costs represent a significant component of healthcare-related expenses, directly contributing to individual financial burden [9]. The observed reduction in financial difficulties suggests a reduction in indirect costs, particularly those related to transportation and caregiver time [3].

In contrast, most symptom-related items did not show statistically significant differences. This observation may be explained by the high burden of comorbidities commonly present in oncologic populations, which significantly impact both QoL and symptom burden. Nevertheless, reducing hospital visits likely contributes to increased patient comfort and decreased exposure to infectious risks [4].

Furthermore, home-based oncology care may represent an important strategy to improve selected aspects of patients' QoL and promote a more patient-centered model of care. In an aging population with increasing cancer incidence and growing demand for systemic treatments, alternative care models such as domiciliary oncology may contribute to more accessible, individualized, and sustainable healthcare delivery. The ONConsigo project demonstrates the feasibility of implementing this approach within the Portuguese healthcare system and provides a basis for further evaluation in larger prospective controlled studies.

Limitations

This study has several limitations that should be considered when interpreting the findings.

One important limitation is the inclusion of both prospectively and retrospectively assessed patients. For a proportion of participants, baseline questionnaires were completed retrospectively after treatment initiation, introducing the possibility of recall and response-shift bias. Consequently, baseline and follow-up assessments were not obtained under identical conditions for all participants, which may have influenced the magnitude of the observed within-patient changes, particularly in subjective QoL domains. Therefore, the observed associations should be interpreted with caution and cannot be attributed solely to home-based oncology care alone.

Another important limitation is the uncontrolled observational design and the absence of a comparator group receiving standard hospital-based oncology care. Consequently, the observed improvements cannot be attributed solely to the ONConsigo program and may also reflect natural adaptation to treatment, regression to the mean, disease evolution, or other temporal factors unrelated to the home-based care setting.

The relatively small sample size may also have reduced the statistical power to detect smaller, although potentially clinically meaningful, changes in other QoL domains. Furthermore, the predominance of patients with prostate and breast cancer may limit the generalizability of these findings to other tumor types and therapeutic settings.

Although the Social Functioning domain was analyzed according to the EORTC QLQ-C30 scoring manual, the remaining EORTC QLQ-C30 and PWI analyses were conducted primarily at the individual item level as part of an exploratory analysis. This approach may limit direct comparability with studies reporting only validated multi-item scale scores.

In addition, the variable follow-up interval (2-6 months) introduced some heterogeneity in the timing of outcome assessment, although no significant association was observed between follow-up duration and the magnitude of change in the EORTC QLQ-C30 items that remained statistically significant after adjustment.

Despite these limitations, this study provides real-world data on the implementation of home-based oncology care and identified several QoL domains in which favorable within-patient changes were observed. These findings should be considered hypothesis-generating and warrant confirmation in larger prospective controlled studies involving more diverse patient populations.

## Conclusions

In this exploratory real-world study, significant differences between baseline and follow-up were observed in treatment-related interference with family life, social life, and financial difficulties. These findings suggest possible associations between home-based oncology care and selected QoL domains; however, they should be interpreted with caution given the absence of a control group and the potential for recall bias.

Overall, this study provides real-world data on the implementation of home-based oncology care in Portugal and supports further evaluation of this care model. Further multicenter prospective controlled studies with larger sample sizes are warranted to better define its impact on patients' QoL, as well as its clinical, economic, and organizational implications, while ensuring appropriate patient selection, robust organizational structures, and strict safety protocols.
